# The heavy-tailed valence hypothesis: the human capacity for vast variation in pleasure/pain and how to test it

**DOI:** 10.3389/fpsyg.2023.1127221

**Published:** 2023-11-16

**Authors:** Andrés Gómez-Emilsson, Chris Percy

**Affiliations:** ^1^Qualia Research Institute, San Francisco, CA, United States; ^2^University of Derby, Derby, United Kingdom

**Keywords:** wellbeing and happiness, pain, valence, emotion, psychophysics, philosophy

## Abstract

**Introduction:**

Wellbeing policy analysis is often criticized for requiring a cardinal interpretation of measurement scales, such as ranking happiness on an integer scale from 0-10. The commonly-used scales also implicitly constrain the human capacity for experience, typically that our most intense experiences can only be at most ten times more intense than our mildest experiences. This paper presents the alternative “heavy-tailed valence” (HTV) hypothesis: the notion that the accessible human capacity for emotional experiences of pleasure and pain spans a minimum of two orders of magnitude.

**Methods:**

We specify five testable predictions of the HTV hypothesis. A pilot survey of adults aged 21-64 (*n* = 97) then tested two predictions, asking respondents to comment on the most painful and most pleasurable experiences they can recall, alongside the second most painful and pleasurable experiences.

**Results:**

The results find tentative support for the hypothesis. For instance, over half of respondents said their most intense experiences were at least twice as intense as the second most intense, implying a wide capacity overall. Simulations further demonstrate that survey responses are more consistent with underlying heavy-tailed distributions of experience than a “constrained valence” psychology.

**Discussion:**

A synthesis of these results with prior findings suggests a “kinked” scale, such that a wide range of felt experience is compressed in reports at the high end of intensity scales, even if reports at lower intensities behave more cardinally. We present a discussion of three stylized facts that support HTV and six against, lessons for a future survey, practical guidelines for existing analyses, and implications for current policy. We argue for a dramatic increase in societal ambition. Even in high average income countries, the HTV hypothesis suggests we remain far further below our wellbeing potential than a surface reading of the data might suggest.

## 1. Introduction

*“Am I not the same being who once enjoyed an excess of happiness, who at every step saw paradise open before him, and whose heart was ever expanded towards the whole world? And this heart is now dead; no sentiment can revive it. My eyes are dry; and my senses, no more refreshed by the influence of soft tears, wither and consume my brain.”* Johann Wolfgang von Goethe, The Sorrows of Young Werther (1774)

The intensity of felt experience has long attracted attention, both academic and lay alike. The quote from Goethe above illustrates how the notion of extreme highs and lows of emotion has captured the imagination of novelists. Sufficiently so in this case that Goethe’s depiction of depression and suicide reportedly led to copycat suicides and the decision to ban his book (Furedi, 2015).

In this paper, we propose a hypothesis that the human capacity for felt sensations and emotions encompasses an incredible range of highs and lows, focusing here on emotional experiences of pleasure and pain. We call this the “Heavy-Tailed Valence” (HTV) hypothesis, named after the early affective-circumplex model of emotions (Russell, 1980) and the feature of heavy-tailed distributions whereby more extreme experiences happen more frequently than casual observation suggests. The hypothesis holds that the most intense pleasures (or pains) are at least two orders of magnitude more intense to experience than the mildest – and that intense experiences are accessible at least in principle sufficiently often that there is policy relevance in considering them.

The HTV hypothesis contrasts against a “constrained valence” hypothesis, i.e., one in which the most intense experiences are no more than 10 times more intense than the mildest or in which any more intense experiences are so vanishingly rare that they can be discounted for practical purposes.

The constrained valence hypothesis is implicitly imposed in many policy interpretations of common measurement scales, such as the single order of magnitude spanned in an integer 11-point scale from 0 to 10. Such scales are common in wellbeing economics, wellbeing policy, and philanthropy (e.g., OECD, 2013; What Works Wellbeing, 2017; Plant, 2019, 2020), despite academic criticism and a call for ordinal-only interpretations (e.g., Bishop and Herron, 2015; Kero and Lee, 2016; Wodak, 2019). The debate on the adequacy of such scales remains contested as of 2023 (e.g., Larroulet Philippi, 2023; Samuelsson et al., 2023). However, the focus of debate is typically around comparability, linearity, and neutrality, rather than the implied human capacity of underlying experience. This paper contributes to the debate by introducing the distinction between constrained and heavy-tailed valence as a related but underexamined issue, by presenting initial evidence for the latter, and by describing practical implications for improved measurement, policy analysis, and policy ambitions implied by an HTV psychology.

The structure of the paper is as follows. The literature review in section two defines our terms within the context of academic work on emotions (§2.1), introduces the debate between cardinal and ordinal interpretation of measurement scales (§2.2), and sets out the conditions under which five empirical predictions can be derived to differentiate an HTV psychology from a constrained valence psychology (§2.3). The method section explains how our pilot survey approach tests two of these five predictions, by asking respondents to compare the most painful and most pleasurable experiences they can recall, alongside the second most painful and second most pleasurable such experiences.

The results are presented in section four, finding cautious support for the hypothesis. For instance, over half of respondents said their most intense experiences was at least two times as intense as the second most intense, which suggests only little extrapolation needed for the full range from the mildest experiences to span at least two orders of magnitude. Simulations also demonstrate a better fit to a heavy-tailed underlying distribution. Our results also raise doubts about the suitability of 0–10 integer scales, at least at the high end, with 81% of users opting for additional granularity when it is available. Over 85% of individuals also describe their most extreme experiences as more intense relative to their second such experiences than would be implied by the scores they place on a 0–10 scale.

In the Discussion section, we first summarize the key findings and explain how they might be interpreted in the context of other research that points toward the sufficiency of a 0–10 integer scale (§5.1). Specifically, we identify the possibility of a “kink” in self-report habits, such that the approximate cardinality of most of the scale, up to around 8 perhaps, may be sustained alongside a compression in reported experiences at the top end of the scale. Secondly, we present two practical implications for measurement techniques and two practical implications for analysts and research funders that would improve policy making in HTV settings (§5.2). We then turn to addressing potential theoretical criticisms of the HTV hypothesis. One concern is that the capacity for experience could arbitrarily be mapped to different scales without any implications for subjective experience. We refute this arbitrariness claim by exploiting the phenomenon of just-noticeable differences in a novel thought experiment: “the integer experience test” (§5.3). Brief accounts are then presented against six stylized facts that run counter to the HTV hypothesis, with three stylized facts presented in its favor (§5.4). Finally, we discuss the limitations and lessons learned from the pilot study, to lay the groundwork for larger scale empirical testing of the hypothesis (§5.5).

The conclusion summarizes the paper and contextualizes it within current data on wellbeing in high average income countries. Unlike the constrained valence hypothesis, our HTV hypothesis leads to a dramatically different interpretation of the current data. Rather than the complacency or incremental improvement potential revealed in the former, we would argue for far greater policy ambition. The future need not be slightly better than the present – it could be almost unimaginably better.

## 2. Literature review

This section first defines our terms in the context of academic work on emotions (§2.1), then introduces the debate between cardinal and ordinal interpretation of measurement scales (§2.2), and finally sets out the conditions under which five empirical predictions can be derived to differentiate an HTV psychology from a constrained valence psychology (§2.3).

### 2.1. Measurements of pleasure and pain

The measurement and taxonomies of emotion remain contested today, with no shortage of alternative theories (Mauss and Robinson, 2009; Ekkekakis, 2013; Keltner, 2019). Nonetheless, in most dimensional models of emotion, there is an axis for positivity or close variants on that theme: pleasurable-unpleasurable (Wundt, 1897), pleasantness-unpleasantness (Schlosberg, 1954), high-low valence (Russell, 1980), and so on.

Emotions at the negative end of these axes have typically not explicitly named pain, perhaps considered more of a sensation than other emotions named on the scales, such as stressed, anxious, fearful, or hostile. However, this may be an omission given developments in the understanding of pain. In a recent cross-disciplinary paper of medical, psychological, and psychiatric experts, Gilam et al. (2020) emphasize that pain is defined as an unpleasant subjective experience with a sensory and an emotional component, although they acknowledge (and regret that) pain has traditionally been researched and clinically treated separately from emotion. Pleasure is similarly not a “pure sensation” (Berridge and Kringelbach, 2008), although externally-stimulated sensations may often be a key input.

In the taxonomy of Dolan and Kudrna (2016), our research focuses primarily on experienced pleasure and pain sensations while acknowledging that some evaluative component remains present, particularly in survey instruments that rely on recollections of past experiences. Such experiential happiness is not easy to capture, but several high-quality methods are available for it. For instance, the Experience Sampling Method (ESM) asks people at random times of the day how they are feeling in the moment. The Day Reconstruction Method (DRM) asks people to recall how they felt during various activities over a 24-h period, which covers a longer period of time but at the cost of overlaying additional memory and evaluative processing of the experiences. Both are burdensome techniques in normal application and if such exercises were to capture rare, peak events, they would need to be asked over a long period of time and most likely in a wide range of circumstances. Such circumstances are unlikely always to be conducive to survey completion, especially considering the complex relationship between emotional intensity and self-awareness (Silvia, 2002), such as may be prompted by taking surveys about your emotions. What we pay attention to in a moment, itself an influenceable phenomenon, is also likely to be important for wellbeing and may differ from judgments about our preferences made in hindsight (Dolan et al., 2021).

In practice, many survey instruments only have space for fewer questions and less frequent surveying, often favoring 11-point integer scales from 0 to 10 for experienced happiness and more evaluative measures of happiness as a result (OECD, 2013; Plant, 2019). Indeed, measurement of pleasure/pain most commonly takes place on short self-report scales using common-sense language (e.g., Haefeli and Elfering, 2005; Dolan and Kudrna, 2016). A 2019 review of Outcome Measures by the Faculty of Pain Medicine (FPM, 2019) presents three pain quantity measures out of 16 instruments related to the topic. It recommends the NPRS, a 0–10 integer scale anchored by 0 “no pain” and 10 “extreme pain/worst possible pain,” over the five-unit verbal rating scale and marking a horizontal line in the Visual Analog Scale.

The discussion of pain in the context of pleasure illustrates the ambiguity in sensations vs. emotions. Some sensations can be mostly separated from emotional content, e.g., experiences of heat, proprioception, or the color yellow may evoke none or several different emotions depending on the context. However, the sensation of pain is almost definitionally valent, whether based on external sensations (nociception), damaged nerves (neuropathy), or system hypervigilancy (nociplasticity). If there is no felt unpleasantness or discomfort associated with a potential pain experience, arguably no pain is being felt.

Our research focus on the capacity to experience adds an additional complication. It may be hard for an individual to understand their personal capacity to experience emotions until having tested those limits or had various uncommon, extreme experiences - or at least witnessed them at close enough quarters to empathize with the participant. We also note reason to believe that the capacity for emotional experience varies from person to person, given psychological instruments to measure such variation at a trait level (e.g., Larsen and Diener, 1987; Bachorowski and Braaten, 1994) and analysis of reported pain sensations in response to the same clinical stimulus (e.g., Wiech, 2016; Fillingim, 2017; Gilam et al., 2020).

### 2.2. Cardinal and ordinal scale interpretation

Survey scales may typically adopt a fairly constrained integer scale, such as from 1–5 or 0–10, but that does not mean the scale has to be interpreted in a cardinal setting, i.e., where an 8 is not just “high” and “much higher than 4” but specifically twice the value of 4 and the gap between 9 and 10 is the same as between 6 and 7. Indeed, many researchers have criticized such interpretation of Likert-style scales, arguing instead for an ordinal-only interpretation (e.g., Bishop and Herron, 2015; Kero and Lee, 2016; Wodak, 2019).

In clinical settings, pain scales often can be productively used with only an ordinal assumption, tracking self-reported progress over time and informing decisions on managing pain severity (e.g., medication or activity restrictions). The latter requires some common usage of language but this can be managed pragmatically by calibrating within an individual patient’s experience over time. Indeed, there is widespread clinical acknowledgement of the limits of inter-personal comparisons given apparent individual variation in pain experience/reporting (e.g., Fillingim, 2017; Gilam et al., 2020).

Unfortunately, in a policy setting, particularly for wellbeing economics, average empirical insights drawn from scales like these are often used in a more strictly cardinal sense (e.g., What Works Wellbeing, 2017; HM Treasury, 2021). The cardinal interpretation of such scales as a reporting function in the context of human communication is commonly applied in practice as a necessary default.

The Happier Lives Institute (HLI) provides a rare theoretical defense of this cardinal interpretation (Plant, 2020). Their account predominantly centers on evaluative wellbeing data (with metrics like life satisfaction). However, there is an aspiration to expand its scope to incorporate additional measures, such as hedonic wellbeing, the focus in our particular scenario. We are unsure that evaluative and hedonic wellbeing are necessarily experienced with the same range of potential capacity, noting differences discussed by Dolan and Kudrna (2016). In particular, evaluative wellbeing may be more constrained at the top end by cognitive and meta-cognitive considerations, such as concerns about future implications or repeatability and what satisfaction is being measured relative to, i.e., what someone might expect or feel they deserved, relative to their personal past experience, identity narratives, and social norms within different communities. However, similar statistical methods as Plant (2020) could be applied to hedonic wellbeing data collected in the future, such as the argument from homoskedasticity of errors.

The theoretical case from Plant (2020) can be applied more directly to hedonic wellbeing without the need for new data collection and analysis. He argues that respondents are likely to interpret a 0–10 scale linearly by default, pointing to analogies with linear scales elsewhere for known cardinal entities (such as distance or income where objective and subjective measurements line up tolerably well), the mathematical difficulty of working with non-linear scales, or the game theoretic consequences of using scales to support effective interpersonal communication (the “Grice-Schelling hypothesis”). However, such rational application may not mean it accurately reflects the range of feeling in the moment, even though we use it afterwards for ease of communication or claim with hindsight how the scale “should” be used. By contrast, the widespread presence of accurately equi-interval metrics elsewhere in society (e.g., measuring distance/weight) might mean we have a tendency to over-impute and rationalize linearity into situations for which it is inappropriate (consider the representational fallacy discussed in Wodak, 2019).

HLI is currently enhancing this theoretical account with empirical data, exploring surveys to test the cardinality of 0–10 scales. Pilot results, maintaining the focus on evaluative wellbeing only, are presented in Samuelsson et al. (2023). The results are tentatively supportive of cardinality but with significant variation and an acknowledgement that more research is needed. For instance, only a slim majority, 56% of participants reported that they used the scale linearly. Most participants said they interpreted the end points of a 0–10 scale as the most extreme possible, split between the most extreme possible for any human and for themselves, but with some inconsistency in answers. However, a substantial minority anchor the reference points in their personal previous experience. They also find (ibid, Figure 15) interesting within-persons variation between their responses on an 11-point and a 10,001-point scale, although the between-persons averages at each of the 11 points line up linearly.

A possible reconciliation of the HTV hypothesis with the HLI account is presented in §5.1, building on the observation that HLI data and principles apply most strongly to common wellbeing experiences, i.e., day-to-day experiences, whereas our current evidence applies only at the extremes.

### 2.3. Contingent empirical predictions of the HTV

The key difference between the HTV and a constrained valence hypothesis concerns the human capacity for experience, with implications both for our understanding of the underlying psychology and for how it is measured. If the most intense pleasures (or pains) are within a single order of magnitude of the mildest pleasures (or pains) or if anything beyond that is discountably rare, then we would describe this as a constrained valence psychology. If the capacity to experience spans a much wider range, say at least two orders of magnitude, and these intense experiences are accessible to us then HTV psychology applies.

The inaccessibility of private experiences means that additional assumptions are needed to differentiate the two hypotheses. Under different sets of assumptions, we can specify five empirical differences between the survey results you would expect if asking people to reflect on experiences sampled either from an HTV psychology or a constrained valence psychology.

#### 2.3.1. Ratio of intense to mild experiences

Focusing first on direct measures of the span of experience capacity, provided individuals have had enough experiences and can call enough of them to mind (even if only in a general sense) to encompass some mild or neutral events and some more extreme events, we would also expect most of them to describe the differences between their least and most intense events as dramatic, whether using narrative or reflecting their intuition as best they can numerically. Where they feel able to use a numerical parallel, most people with diverse life experiences should describe this span as more than 100x, i.e., two or more orders of magnitude.

#### 2.3.2. Ratio of intense to average and average to mild experiences

Similar principles can be applied using the average point instead of a neutral point. The ratio of intense to average should be higher than the average of average to mild. This approach is less sensitive to identifying the most extreme and most neutral experience and it may be easier for respondents to reflect on an average experience as a reference point. However, it is a less direct measure of the actual span of experience.

#### 2.3.3. Ratio of most to second most extreme experiences

Provided individuals have not had so many experiences that the full spectrum of possibilities is filled in, we would also expect larger differences between their most extreme and second most extreme memories. If you sample only 10 experiences first from a 10-step scale and secondly from a million-step scale (where the scales here linearly reflect the true underlying range of experience), using any identical distribution that spans the full range of experiences, whether via a uniform, normal, or heavy-tailed distribution, the ratio of the largest to the second largest will, on average, be much larger if sampled from the million-step scale. Whereas if a million or more experiences were sampled, this ratio can arbitrarily approach one on any continuous distribution. Provided the number of experiences sampled and recalled for the purposes of the comparison remains well below such levels, we arrive at a meaningful ratio. The stated ratios can then be extended to identify the implied number of equivalently sized steps to span at least two orders of magnitude from the top to the bottom end. If this number of steps feels intuitively low compared to the range of experience that actually exists or is reported as such by participants, it is indirect evidence for the HTV.

The previous three methods test the pragmatic relevance of the upper ends of intensity indirectly, in that if survey respondents are remembering them then we infer that the events are likely occurring with sufficient frequency to be relevant. In other words, vanishingly rare events would also be vanishingly rare in our survey data. As an aside, this implies that rejections of the HTV using these tests cannot differentiate between rejections based on span size as opposed to extrema accessibility. It is also possible to test distribution of experience more directly.

#### 2.3.4. Distributional fit

All else being equal, a heavier tailed distribution will, by definition, have more extreme events more frequently than a narrow-tailed distribution. Indeed, a narrow-tailed distribution is one of the phenomena that could mean a high capacity to experience nonetheless translates in practice to a constrained valence psychology. A narrow-tailed distribution is likely unable to model the range of experiences in the majority of moderate, quotidian experiences while still preserving enough likelihood for outlier events two or more orders of magnitude out.^1^ Distributional fit could be tested directly on valence data across individual experiences or indirectly on which underlying distributions would produce other metrics, such as the ratios specified in the first three methods. A limitation of this method, unlike the first three, is that the hypothesis is not yet explicit on how accessible extreme events need to be for relevance and so it is unclear how heavy-tailed a distribution needs to be. However, canonical distributions that are commonly approximated in nature could be used as initial reference points, such as the normal distribution (generated, e.g., via additive input processes by central limit theorem mechanisms) compared against the lognormal distribution (generated, e.g., via multiplicative input processes).

From an HTV perspective, recollection-based survey approaches should underestimate the true capacity to experience, as it is highly likely that most people’s best and worst actual experiences by a particular date are not the best or worst experiences that are accessible in principle - or even the best or worst that they will have experienced by the end of their lives.

#### 2.3.5. Direct inquiry

Finally, for completeness, it is also possible to ask people directly about the most intense and mildest experiences they can imagine and to comment on the span between them and what it might take to access different states.

Across all these methods, where we are analyzing responses from a sample of individuals, we also need to assume that differences between individuals in terms of how they report emotions and differences in terms of the underlying capacity to experience are either not correlated or at least only modestly biased with respect to the range being analyzed.

## 3. Methodology

We conducted a pilot survey to test two of the five empirical predictions from §2.3. This section explains the survey design (§3.1), the analytical methods that allow the survey question data to be related to the empirical predictions (§3.2), and finally the survey implementation, data cleaning, and participant demographics (§3.3).

### 3.1. Survey design

The survey is designed to compare the most and second most extreme experiences recalled, rather than average or mild experiences, reasoning that the assumption on modest numbers of recallable peak experiences is more plausible than assumptions on the reliability of assessing mild or average experiences. Further, a setting in which someone is more easily able to recollect peak pleasurable experiences may report their average experience as more positive, setting up a confounding correlation in the analysis of interest. It may also be hard to identify an average experience as an abstract idea and it might be heavily affected by recent activity. Whereas by asking respondents to focus on specific events, we are more confident they have specific emotions in mind. For the same reason, we did not prioritize the direct inquiry method, noting also that it elides differences between the capacity to imagine and capacity to experience, and that the under-estimate approach of recollection surveys helps build in a conservative methodology that would, all else being equal, increase confidence in HTV-positive findings.

We asked respondents to state what their most, second most, and third most pleasurable experiences were, to explain what category they fell into, and to write a short account of the most intense experience, with identical questions for the most painful experiences. With respondents having these experiences in mind, we asked them to use a slider to rate each one on a scale from zero to ten, from no pleasure/pain to the highest possible pleasure/pain. Non-integer responses were allowed out to a single decimal point. Illustrative descriptions were placed along the scale to support interpretation, anchoring the null experience at “0” for none, followed, e.g., by “1. Slightly bad feeling,” “3. The pain is bothering me but can be ignored,” and “8. The pain is so intense it is hard to think of anything else.” These scalar questions allow us to contrast against standard measurement scales used.

The main novel question that generates traction on the HTV is then asking for free text estimates of the ratio: “Relative to the 2nd most painful experience, how many times worse was the single most painful experience in #1?” This paper refers to this question as the “described ratio” question. Respondents may have been primed by first providing the scalar responses, such that a numerical intuition for cardinality and consistency over-rides a true reflection on the felt experience, e.g., calculating that the ratio should be 1.25x as they have already provided a 10 and an 8 for the scalar questions, even if 1.25x underplays their felt experience. However, this bias was tolerated on the same basis as the recollection approach: it errs toward a conservative methodology that disfavors the HTV.

In designing the analysis, we wanted to allow individuals to think in terms of intensity of experience, accepting that different angles on pleasure or pain may have different intensities for different people. Some may think more of blissful joys, others more of adrenaline-filled thrills; some may focus on heartbreak, others on physical pain. It is debatable how much pleasures and pains with different sources and inflections are directly comparable, but nonetheless it is often possible to comment on which is more intense and whether it is much more or only slightly more intense, suggesting that many of us have some internal mechanism for forging quantitatively-nuanced comparisons even if the underlying experiences are multidimensional. Such mechanisms are required and implied by methods such as willingness to pay, time trade-off, and standard gamble questionnaires used in health economics (e.g., Lipman et al., 2019).

We also asked about current age and age the experiences happened, gender, current feeling of pleasure/pain, and which of their most extreme pleasure or pain was more intense.

### 3.2. Analytical approach

In addressing our primary question, the likely presence of HTV psychology in the sample, we use two analyses based on the options set out in §2.3. The first examines the intensity ratio between most and second most intense experience, as described directly by respondents, considering pain and pleasure responses separately. As well as examining the described ratios descriptively, we consider how many similarly sized steps would need to be present at minimum for felt experience capacity to span two orders of magnitude on its way to the mildest experiences.

Secondly, we conduct statistical simulations to examine whether the described ratio responses are a better fit to an underlying set of experience valences that are normally or lognormally distributed, as example canonical narrower and heavier tailed distributions.

As secondary analysis, the survey questions also permit some insight into how users might be engaging with traditional 0–10 integer scales, an important related measurement question. We report also therefore on how many respondents made use of decimal points and how many users gave ratio responses that were consistent with their scalar scores, as would by implied by cardinal use of a 0–10 score with 0 as the neutral point, as requested in the notes to the user.

### 3.3. Survey implementation, sample selection, and participants overview

We ran the survey on Mechanical Turk in 2019, with respondents receiving US$ 1.75 for completing the survey. In addition to the implicit inclusion criteria (English speakers with access to the platform), there were two explicit inclusion criteria for responding to the survey: a good track record of task completion on Mechanical Turk and a master’s qualification, both designed to increase the chance they would engage with the questions. Funding approval and operational/ethical sign-off were provided by the Qualia Research Institute leadership team, noting that the survey was opt-in, open only to adults on an anonymous basis, and reimbursed with a modest contribution to thank them for their time. The request to reflect on emotionally challenging experiences was seen as balanced by the request to reflect on pleasurable experiences, and by the focus on highly-educated respondents more likely accustomed to exploring challenging topics via questions and essays.

The initial 110 responses were analyzed to remove 13 likely bots (or non-serious completions), based on non-sensical or off-topic responses to the mini essay questions. Further exclusions or adjustments were also applied for responses that had ambiguous interpretations or were not mathematically consistent. Given uncertainty in this process, we tested the analyses against two types of sample. First, against smaller but clean samples having removed any ambiguous or conflicting answers. Secondly, against the maximum possible sample based on interpreting ambiguous answers in the direction that is most disfavorable to the HTV, in line with our other conservative methodological choices. For instance, a reported “1x” difference between the most and second most intense experiences would conflict with non-identical scalar scores. The clean sample excludes such responses but the maximum possible sample applies the HTV-disfavorable interpretation that the 1x is a valid response (i.e., no difference), assuming that the respondent’s differences on the scalar questions are felt to be of negligible importance to them.

The full detail of exclusions and interpretations is shown in Table 1. For the primary analyses focusing on the described ration questions, the maximum HTV-disfavorable sample is 91 for the topic of pain experiences and 95 for pleasure experiences, i.e., sample exclusion rates of 6% and 2%, respectively. The clean sample for described ratio analysis is 77 for both, i.e., a sample exclusion rate of 21%. Secondary analyses contrast against the scalar responses, i.e., the 0–10 intensity scores, for which the clean samples are 65 and 64, respectively.

**Table 1:** Exclusions to generate clean samples for analysis.

Sequential exclusion steps for described ratio analyses		Sample size	
#	Exclusion detail	Pain responses	Pleasure responses
1	Full initial sample	110	110
2	Likely bots or non-serious completions, based on non-sensical or off-topic responses to the free text questions	97 (−13)	97 (−13)
3	Those who provided described ratio text that could not be interpreted as a number, e.g., “much worse”	96 (−1)	96 (−1)
4	Those reporting very high ratios of 100x or higher that perhaps have a narrative interpretation of “much much more intense” but risk being misleading if interpreted mathematically at face value (the HTV-disfavorable interpretation is to exclude such outliers)	91 (−5)	95 (−1)
5	Those who reported a “zero times” ratio difference or equivalent (interpretable disfavorably as the experiences being the same, i.e., 0x would be coded as 1x)	88 (−3)	93 (−2)
6	Those who reported a ratio difference between zero and one (e.g., 0.5), which mathematically implies that the second most extreme was more extreme than the most (only interpretable as the given ratio being on top of the original, e.g., 0.5x would be coded as 1.5x the original)	85 (−3)	86 (−7)
7	Those reporting “one times” difference or equivalent but whose reported scalar scores were not identical or within 0.4 points (interpretable disfavorably as 1x, although they might have meant one times better/worse on top of the original experience)	77 (−8)	77 (−9)
8	For separate analyses comparing the descriptive ratio responses against the scalar 0–10 question responses, we further exclude those whose scalar responses reveal a mathematical misunderstanding, i.e., the second most intense experience is scored as more intense than the most	65 (−12)	64 (−13)

Table 2 reports the sample demographics, identifying a near even gender balance and a wide age range, broadly consistent across the different analytical samples.

**Table 2:** Sample demographics.

Demographic aspect	*N*	% Male^1^	Age range	Age mean (st. dev.)
Maximum HTV-disfavorable sample (pain)	91	49%	21–64	37 (10)
Maximum HTV-disfavorable sample (pleasure)	95	48%	21–64	37 (10)
Clean described ratio sample (pain)	77	47%	21–64	37 (10)
Clean described ratio sample (pleasure)	77	48%	21–64	37 (10)
Clean comparative sample (pain)	65	51%	21–60	37 (10)
Clean comparative sample (pleasure)	64	48%	22–64	38 (10)

^1^Note that all respondents self-described as either Male or Female (free text box).

## 4. Results

### 4.1. Capacity estimation given described intensity ratios

Table 3 reports descriptive statistics for the described ratios between the most intense and second most intense experiences, for both the maximum HTV-disfavorable sample and the clean described sample as defined in Table 1, 2. The results suggest a wide range of described intensity ratios, from effectively no difference between most and second most intense experiences (i.e., ratios of 1x) to those suggesting far more dramatic differences (e.g., ratios of 5x+).

**Table 3:** Described ratios of most intense and second most intense experiences.

Descriptive statistics	Maximum HTV-disfavorable sample	Clean described ratio sample
*Pain responses*		
N	91	77
Range*	1.0–50.0	1.0–50.0
Mean (standard deviation)	4.9 (9.1)	5.6 (9.7)
Median (interquartile range)	2.0 (1.2–5.0)	2.0 (1.5–5.0)
*Pleasure responses*		
*N*	95	77
Range*	1.0 - 99.0	1.0–99.0
Mean (standard deviation)	5.1 (13.8)	6.0 (15.2)
Median (interquartile range)	2.0 (1.1–3.0)	2.0 (1.5–3.5)

*i.e., 1x would mean the most intense and second most intense experiences have effectively the same intensity for that respondent; 1.1x would correspond to 10% greater intensity, e.g., a 9.9 on a truly cardinal ratio scale compared to a 9.

The median is 2x for both samples and both pleasure and pain. In other words, 50% of respondents describe their most intense experience as 2 or more times as intense as the second most intense. It would require around six equivalently sized ratio steps between the mildest and most intense possible experiences to support the two orders of magnitude in the HTV. Within the clean sample 75% or more respondents identify an intensity ratio of 1.5x or higher, which would translate into 10 equivalently sized steps. Even ratios of only 1.1x-1.2x would only require some 30 equivalent steps.

We note also that the sample exclusions in steps 3 and 4 of Table 1 correspond to responses that would strongly endorse an HTV psychology. All of these responses (6 for pleasure; 2 for pain) described dramatic differences between the most and second most intense experiences, such that it would require very few further steps (none, in some cases) to span two orders of magnitude. Even if these numbers are best interpreted qualitatively rather than quantitatively, they point to strongly felt differences in the respondents.

### 4.2. Statistical simulation of underlying valence distributions

Subsequently, we evaluate the described ratio responses by comparing them to simulations based on an assumed underlying valence distribution of experiences, choosing either a normal or log-normal latent distribution as examples with differing kurtosis. As discussed earlier, extrema ratios will approach one as the simulated individuals are assumed to isolate individual experiences across a larger number of recalled experiences. We simulate three scenarios reflecting different such numbers of recalled experiences (10, 100, and 1,000 experiences per individual), with 1,000 simulated individuals in each scenario. This part of the analysis is presented only for the described ratios for the pleasure questions (*n* = 77), because the simulations lead to the same conclusions for the pain questions, as expected given the high similarity between the two distributions (Table 3).

The described ratio distribution we are analyzing is the ratio of two extrema - the largest and second largest an individual can recall - rather than the underlying distribution of individual valence experiences. As a result, direct measures of tail heaviness (such as kurtosis) or standard measures of normality are not applicable. Instead, the extrema statistics end up individually distributed in the limit according to the GEV (generalized extreme value) distribution, regardless of the underlying distribution (provided regularity conditions are met; see Haan and Ferreira, 2007). A convenient outcome of this process is that the standard deviation parameter chosen for the normal distribution comparison does not affect the quality of fit for the emergent extrema ratio distribution. Any positive standard deviation parameter chosen for the underlying normal distribution of experiences translates into the same distribution of extrema ratio (all normal distributions have kurtosis of 3), so we can choose a standard deviation of one without loss of generality and without a need for an optimization process to identify the best fit.

For the lognormal distribution, larger standard deviation parameter choices result in larger ratios in the subsequent extrema ratio distributions. The plotted data therefore compare a standard deviation of one in both cases as sufficient to demonstrate the better fit of the lognormal distribution, noting that a distribution with higher variance, skew, and kurtosis can easily be generated in the lognormal case to improve the fit yet further if desired. A mean of 5 is entered into both normal and lognormal distributions, to ensure that no extrema are negative in the former case. Our simulations suggest the distributional shapes are not sensitive to different choices of sufficiently positive means (only the y-axis range varies), provided the extrema ratio are always based on analyzing two positive numbers.

Finally, we normalize each distribution so it maps to a 0–1 scale, noting the arbitrariness of any maximum unit selection for the same underlying feature of reality, so that all three distributions can be plotted on the same rank-ordered chart for ease of comparison. The two simulated distributions are downsampled to n = 77 to match the applicable survey sample size for the same purpose, using equally spaced multiples of 13 from the size-ordered distribution of 1,000 simulated individuals plus the top extremum. This normalization retains all important parts of the distribution for our purposes because the actual numerical start and end points of the scale are arbitrary in any distribution. Post-normalization, rank ordering and relative centered size of individual data points are the same; skewness, and kurtosis features are unchanged; standard deviation changes only by the scaling parameter.

**Figure 1:**
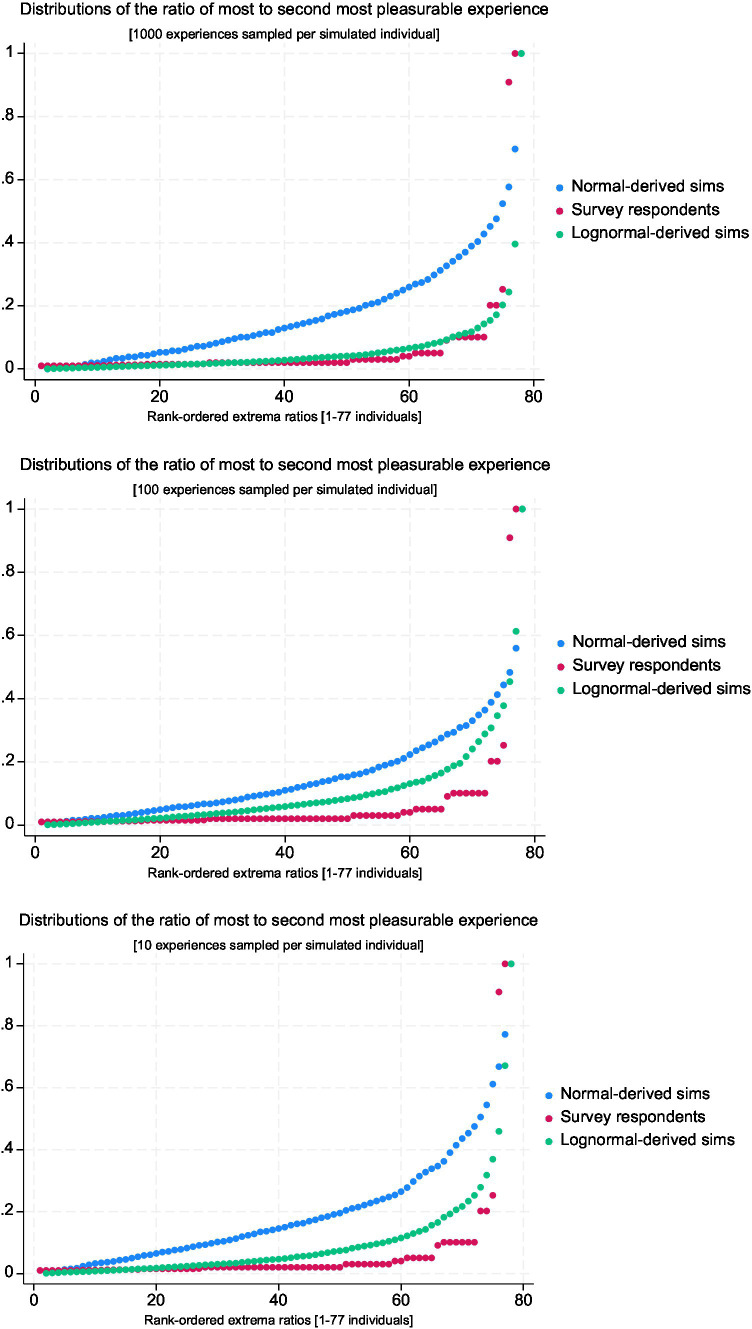
Survey respondents vs. thin/heavy tailed simulations – three scenarios.

Figure 1 shows the three resulting distributions plotted on one chart for each scenario, reflecting different numbers of underlying experiences being sampled by individuals. In all three scenarios, the lognormal-derived distribution (green) is a closer fit to the survey respondents (red) at virtually all points in the distribution than the normal-derived distribution (blue). In the case where 1,000 experiences are sampled, the lognormal-derived distribution is a particularly close fit, suggesting that this particular parametrization may be worth investigating in future work.

### 4.3. Secondary analysis: assessment of 0–10 scale

Table 4 provides descriptive data on the scalar 0–10 intensity scores provided by respondents in the clean comparative sample. The majority of respondents appear to be anchoring their most intense historical experiences as fairly close to the most intense possible, with 75% identifying their most intense pain at 8.8 or higher and their most intense pleasure at 8.9 or higher. While a few people provide low end scores, the vast majority of our data is around 8–10 on the self-report 0–10 scale.

**Table 4:** Intensity scores from the clean comparative sample.

Descriptive statistics	Most intense scores	Second most intense scores
*Pain responses*		
*N*	65	65
Range	3.0–10.0	1.0–10.0
Mean (standard deviation)	9.1 (1.3)	7.7 (1.8)
Median (interquartile range)	9.6 (8.8–10.0)	8.0 (7.0–9.2)
*Pleasure responses*		
*N*	64	64
Range	7.5–10.0	4.6–10.0
Mean (standard deviation)	9.3 (0.7)	8.4 (1.3)
Median (interquartile range)	9.6 (8.9–10.0)	8.5 (7.5–9.4)

Unlike common 0–10 scales, which permit only integer responses, our sliding scale allowed respondents to select intensity scores to one decimal point. Out of the 97 non-bot responses, 81% of respondents used a decimal value in at least one of the four scalar responses (two each for pleasure and pain). 57% of all such scalar responses did use a decimal value. Indirect insight on the importance of such additional gradations comes from how frequently such gradations are used since they permit in theory the cardinal mapping of the 0–10 scale to two orders of magnitude rather than one. This insight is indirect only as it does not guarantee that users are engaging with the scale on such a basis. A safer conclusion is that the majority of respondents prefer to use the additional gradations where they are present and that an integer scale is therefore potentially missing or eliding information about the respondent’s felt experience.

**Figure 2:**
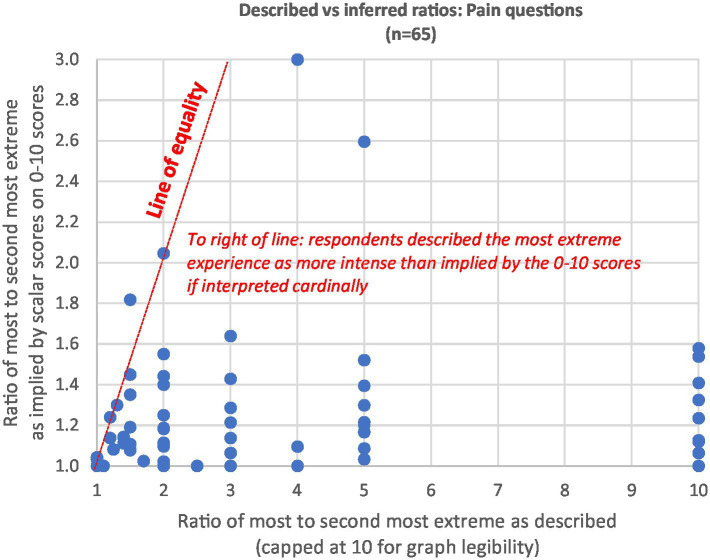
Described vs. inferred ratios: pain questions.

**Figure 3:**
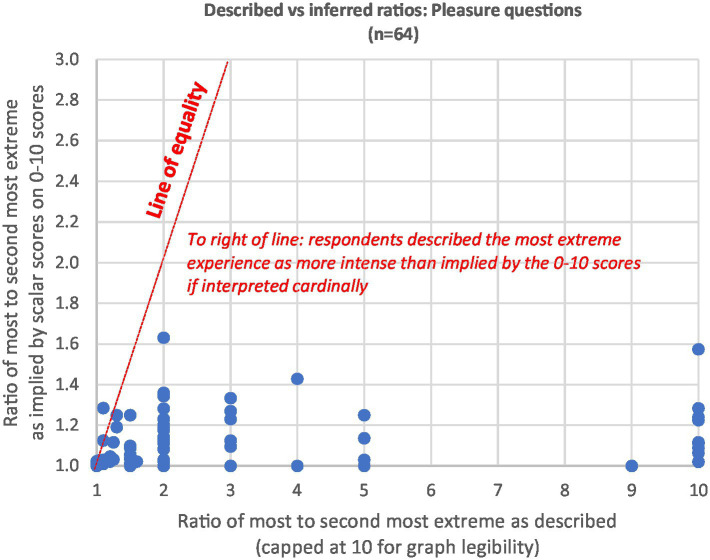
Described vs. inferred ratios: pleasure questions.

A more important assessment of 0–10 scale cardinality comes from comparing the described ratios with the inferred ratios. If the 0–10 scale is intended approximately cardinally by users with a zero neutral point, then dividing the most intense experience score by the second most intense should give a similar answer to the description they give when asked to do so directly. Figures 2, 3 plot each respondent for pain and pleasure responses, using the clean comparative samples from Table 1. The described ratios are strictly larger than the inferred ratios for 88% of respondents on the pleasure experiences (*n* = 64) and 86% on the pain experiences (*n* = 65). They are 1.5x higher or more for 59% of pleasure experiences and 65% of pain experiences.

## 5. Discussion

This section first summarizes the key findings and explains how they might be interpreted in the context of other research arguing for the sufficiency of a 0–10 integer scale (§5.1). Secondly, we present practical implications for measurement techniques, analysts, and research funders (§5.2). We then turn to addressing potential theoretical criticisms of the HTV hypothesis, a concern that capacity for experience could be arbitrarily mapped to difference scales (§5.3) and stylized facts that run counter to the HTV hypothesis (§5.4). Finally, we discuss the limitations and lessons learned from the pilot study, to lay the groundwork for larger scale future testing of the hypothesis (§5.5).

### 5.1. Summary of findings and literature synthesis

The two primary analyses in the pilot survey cautiously support the HTV claim that our capacity to experience pain and pleasure spans at least two orders of magnitude. In the first analysis, 50% of respondents describe their most intense experience as 2 or more times as intense as the second most intense. As adults presumed able to draw on a range of recalled life experiences, it is hard to maintain such a difference in intensity within a constrained valence psychology. It would only require around six equivalently sized ratio steps between the mildest and most intense possible experiences to support the two orders of magnitude in the HTV. Within the clean sample 75% or more respondents identify an intensity ratio of 1.5x or higher, which would translate into 10 equivalently sized steps.

In the second analysis, simulations demonstrate that a canonical heavy-tailed distribution of underlying experiences fit these described intensity ratio data far better than a narrow-tailed distribution. In other words, intense experiences, such as those whose descriptions imply a broad capacity to experience, are more frequent and more accessible in lived experience than would be likely under narrow-tailed distributions. This suggests that such experiences are not so vanishingly rare that they can be pragmatically discounted. Moreover, our method is expected to underestimate the full range of the capacity to experience since respondents are asked to reflect on their actual remembered experiences. Many respondents, particularly younger ones, will on average have had a narrower range of actual experiences than are physically possible. We hope, for instance, that none of our respondents have experienced illegal torture.

Our data also question the suitability of a 0–10 integer scale for understanding intense experiences. Over 85% of respondents describe their most extreme experiences as more intense than their second most extreme experiences than implied by a cardinal interpretation of the 0–10 scale responses. 81% of respondents made use of decimal values in at least one of the four applicable questions, suggesting that they appreciated the additional granularity beyond the 11-points on an integer scale and that there might be valuable information to gain from such granularity.

Other research, discussed in section two, has argued however that an integer 0–10 scale can be interpreted cardinally in most practical circumstances, i.e., a single order of magnitude is sufficient (Plant, 2020; Samuelsson et al., 2023). Rather than take our evidence and the HTV as necessarily rejecting these claims, we suggest a reconciliation of the evidence with actionable implications for policy analysis.

Our synthesis suggests that non-linear interpretation of the 0–10 scale may only happen at the top end. Given that our survey focused on extreme experiences, our evidence by definition is restricted to the top end of the intensity scale for both pleasure and pain, with mean scores of around 8 or above.

People may broadly apply a linear interpretation (as required by Plant’s Grice-Schelling hypothesis for effective communication) for most of an 11-point reporting scale, with 0 being a near neutral experience and 10 being the most intense they imagine is physically possible, up until perhaps 8 or 9. Between 9 and 10 there may be an additional order of magnitude or more of compressed experience, but most people may not have yet experienced the peaks and may even be unaware how much capacity is there. For everyday communicative purposes, all we really need to know is that 9 is already remarkably intense and that 10 is “even higher” since there is nothing above the 10. Indeed, for many policies governments are concerned with today, it may be adequate to consider insights up to 8 or 9 on the pleasure scale. For the pain scale, however, this is less comforting. This compression at the top end of the scale can be described as a “kink” in the scale.

A kink effect could reflect a general phenomenon in which reported outlier experiences are typically compressed when placed on a scale without significant prior thought and analysis, consistent with either the log or arc-tan proposals from Ng (2008). One possible mechanism to generate this is as follows. Someone may have already used large parts of the scale to convey the fact that moderately intense experiences felt much more dramatic than mild ones, hence needing much larger scores on the integer scale for the numbers to align with felt intuition. As they turn their attention to more extreme experiences or the most extreme they can imagine, perhaps with prompting to help appreciate the full range of possible scenarios, they may find there is too little space left on the scale to capture the full range. To do so as best they can, without changing prior answers anchored from other discussions or first attempts to calibrate the scale to quotidian experiences, they have to compress the distance in each point reported as they approach the extremes. This is similar to a child counting from zero to ten for a task to be ready and realizing they need to add nine and a half, nine and three quarters, and so on to create extra time toward the end.

### 5.2. Implications for policy-focused analysis under the HTV

Our key claim is that felt experience intensity spans at least two orders of magnitude. This matters for measurement because many scales either assume a single order of magnitude or permit only ordinal interpretations. Under our hypothesis, there are four implications for improved policy-focused analysis, two focused on measurement and two on the analysis environment.

Measurement techniques should favor pleasure/pain scales with at least 100 and preferably 1,000+ gradations. For instance, if the currently common 0–10 scales are used, it should be possible to report a 7.8 or 9.3 rather than just 8 or 9 and this flexibility should be conveyed explicitly to users. Alternatively, a 0–1,000 scale might be presented directly. If visual analogs or “sliders” are used, they should also be sensitive to such levels of granularity.

Secondly, users providing measurements on scales should be advised about the location of a specific neutral, non-negative point, i.e., what number corresponds to an absence of any pleasure or pain sensations (perhaps 0). Similar to our pilot survey, several reference points should be provided along the scale with vivid corresponding adjectives and types of experiences. Current scales often state only the “best [or worst] possible,” which conveys little about the scalar variation or the intensity of extreme experience, especially given the bland mindset that might prevail during administrative tasks like filling out surveys.

For analysts working with the integer data scales around a single order of magnitude that are common today, ordinal analysis techniques should be preferred. Where such techniques are insufficient, e.g., for comparative intervention trade-offs or cost–benefit analyses for policy/funding reasons, analysts should test the sensitivity of their conclusions to “kinks” in the measurement scale. For instance, testing whether results would still be valid if 8–10 corresponded to as large a change in felt experience as 0–8 on a 0–10 scale. Many policy interventions are analyzed based on arithmetic averaging of self-report data. To be valid in a kinked scale setting, the data need to be first weighted (i.e., with higher weights at the top end) before averaging and entering into subsequent analyses. For instance, to the extent that policies are based on the arithmetic average of pain scales, we can expect an increase in the priority of especially painful conditions once HTV is taken into account.

Finally, researchers and research funders/policy-makers should consider research that might explicitly test for kinks or non-linearities in 0–10 integer scales. For instance, trade-off survey techniques on medical conditions used to define QALYs and DALYs could also be applied to wellbeing questions (e.g., Lipman et al., 2019). Such “willingness to trade” surveys could test, for instance, how many reasonably pleasant days (5–7/10) someone might be willing to trade down to bland days (2–3/10) in exchange for one more “best day you have ever had/could imagine” (9–10/10). Insights on time consistency and intensity/duration trade-offs are needed to interpret such data (see, e.g., McAuliffe and Shriver, 2022) but building a body of such evidence nonetheless helps put parameters around the potential non-linearities that might be present in common survey reporting.

### 5.3. A counter to the arbitrariness critique

The HTV is contrasted against constrained valence in a phrasing that suggests there is a real difference in human experience. It suggests that we can imagine two different worlds, one where it is true and one where it is false, and it *feels* different to exist in either of those two worlds. It is not just that the hedonic rulers have different markings. One critique is to accept HTV but to restrict its relevance just to the design of measurement techniques: the number of gradations on a scale and how the scale is interpreted by users, rather than anything real about human experience. This critique might point to issues like the subjectivity of emotion (e.g., the unknowability and incommunicability of how your “worst pain ever” compares to mine) or the arbitrariness of unit selection (e.g., objects measured to the nearest millimeter span more orders of magnitude than measured to the nearest meter, but the real lengths are unchanged).

The “integer experience test” thought experiment refutes the arbitrariness critique by imagining an environment where one could toggle the HTV hypothesis on and off for a single experience, e.g., for a few minutes or hours engaged in a single activity, showing that the test participant both feels and reports genuine differences depending on whether the HTV setting is activated.

The thought experiment requires two core assumptions. The first assumption is that there is something to remark on. There are at least some experiences that vary in the pleasure or pain intensity evoked in an individual. This does not remove the possibility of multiple other dimensions that affect the emotional response, nor does it deny the possibility of complex, mixed-valence experiences (Gómez-Emilsson, 2022), and nor does it require the reaction to be the same across individuals or over time. The second assumption is that we do not have infinite sensitivity, i.e., that current human systems cannot perfectly identify differences at the smallest imaginable level of tweaking pain or pleasure. For instance, our nervous system is not perfectly sensitive. We can also imagine tiny numerical differences in scenarios that would not meaningfully alter the joy we experience from them (e.g., a life extended by a single femtosecond or a large lottery win extended by a single cent).

In this hypothetical test, a participant begins in a neutral state with no/negligible pain (or pleasure) being experienced. We expect most readers have experienced or can imagine such a neutral state.^2^ Trivially small increases in pain are added into the experience, gradually increasing until the participant consciously reports noticing a difference, however small. That marks a single integer step or score, drawing on the principle of just-noticeable or least perceptible differences in psychophysics as commonly applied to direct sensory dimensions such as light brightness or sound intensity/pitch (e.g., Kollmeier et al., 2008). The process continues, continually marking integer steps with every consciously reported difference by the participant. While there would likely be very many such steps in any scenario, in an HTV there would be many more such steps – a higher score - before the human system loses consciousness or ceases to notice any difference no matter how much pain is added.

The same process can be done separately for experiences of pleasure, e.g., perfect brain simulations of different scenarios and sensations that elicit pleasure responses, noting many possible contributing factors in such scenarios (e.g., Gilam et al., 2020). If, at some point, additional pleasure experience translates (for any reason) into discomfort or unhappy emotions that the experimenter cannot correct for in the scenario, then we would conclude the pleasure capacity has been capped at the prior number of integer steps. Again, there would be more steps reported in the HTV scenario than a constrained valence scenario. Both the felt experiences and the reports of that experience differ between the scenarios.

The “integer experience test” is independent of any particular measurement scale or a participant’s emotional sensitivity. It is independent because the test is intra-individual, i.e., the participant compares their experience in one hypothetical world vs. their experience in another. It cannot be implemented as a physical experiment, but as a thought experiment it can be presented against other theory-only critiques such as the arbitrariness critique.

### 5.4. Counters to stylized facts against the HTV

Our research and discussions have identified six stylized facts that might be levied against the HTV: (i) action potential phases; (ii) decreased sensitivity at extremes; (iii) hedonic adjustment; (iv) diminishing returns to scale in economic analysis; (v) behavioral change predictions; and (vi) an argument from evolutionary efficiency. Each is presented briefly below with a brief counter-argument.

(i) Many cells that are central to human conscious experience have action potential phases, notably neuron cells in the brain as well as the plasma membranes of most cells (e.g., Purves et al., 2012). The course of the action potential has several phases: the rising phase, the peak phase, the falling phase, the undershoot phase, and the refractory period. The last phase is relevant for this argument and corresponds to the period when subsequent action potential is very difficult or impossible to initiate. In other words, a forced pause after excitation. This places a biological limit on how frequently neural patterns can fire in a period of time. However, while this phenomenon may place a limit on conscious experience insofar as it is mediated by neural patterns, it says nothing about where this limit may be – and how vast a range of experience it might demarcate.

(ii) The decibel scale is usefully logarithmic because human perception of sound intensity more closely responds to the logarithm of intensity, instead of its linear value. Effectively, the human ear reduces its sensitivity as the sensory input increases. If a similar principle applies to valence, it might suggest modest intervals of increased experience even as the drivers of that experience increase exponentially. However, it is also possible that we may only be able to definitely tell that a pain has got worse when increased by a constant percentage, but that does not mean that increased pain units below that threshold are not still unpleasant, it is just that we are so overwhelmed by the volume of pain it is hard to be sure. Sensory data is additionally only one of several inputs to emotional intensity (Gilam et al., 2020).

(iii) The hedonic treadmill is the claim that humans return to previous and relatively stable levels of happiness (“the happiness set point”) following major experiences (Brickman and Campbell, 1971; Perez-Truglia, 2012). Various mechanisms have been proposed, e.g., assessing value against our past memories, evolutionary motivation to set new baselines and keep driving for improvement, neurochemical desensitization, or “abundance denial” given pressures of personal or social identity (e.g., Solomon and Corbit, 1974; Ahmed, 1998; Easterbrook, 2004; Rivat and Ballantyne, 2016). However, the empirical evidence on the hedonic treadmill is contested (Diener et al., 2006; Gardner and Oswald, 2007) and competing mechanisms can exist, such as when increased exposure can increase the joys experienced (e.g., connoisseurs of food or wine; Frederick and Loewenstein, 1999). Nonetheless, even if true, these arguments refer to adaptation or desensitization over time, rather than critiquing the possibility of dramatic experience in the present moment. This may have consequences for the political or personal implications of HTV but not for its truth as a description of our psychological capacities.

(iv) A more direct claim for adaptation within a single experience may come from the diminishing returns to scale applied in many economic models of human preferences and behavior. For instance, for most consumption goods, economists have long typically observed that gaining each additional unit reduces the utility we expect from the next unit and our corresponding willingness to pay (e.g., Gossen, 1854). If it becomes harder and harder to increase valence as individuals either suffer more or experience more pleasure, there may be practical limits to such experiences – although these limits could well be far off from everyday levels today.

(v) Indirect evidence to support the naïve interpretation of linear scales can be found in Kaiser and Oswald (2022), who show that self-reported dissatisfaction with various aspects of life is approximately linearly correlated with the probability of trying something new in that aspect of life. However, there is no compelling reason to believe that satisfaction with someone’s job, house, or partner would be experienced with the same capacity range, or adequately reported on the same sort of scale, as emotions of pleasure and pain, nor that the probability of action should be linearly correlated to underlying emotions. Indeed, as emotions become felt more extremely, it is plausible that other parts of the mental machinery may attempt to dampen down the urgency to act in the present, so that the pros and cons can be weighed up in a more cautious, future-oriented frame of mind.

(vi) Feelings of pleasure and pain play an important function in improving our chances of surviving, reflected in brain structure and functionality (Brehm, 1999; Berridge and Kringelbach, 2008). Plant (2020) notes that processing and experiencing sensations is costly in terms of energy, reflected also in points around brain structure frugality for pleasure sensations. If we assume more intense sensations are more costly, then there is an evolutionary incentive to make our sensations and subsequent emotions only just intense enough to drive us toward action, with enough bandwidth to weigh up an appropriately broad range of options (noting that “wanting to repeat something” and “liking the experience” are related but not identical constructs). While it is unclear how much bandwidth would be needed to reflect the high dimensionality of options that the human system might face, this is an argument that urges toward more tightly bounded capacities. A counter argument would note that in the ranges of normal circumstances and behavior – presumably the ranges that evolution primarily incentivizes for - there may be many individual factors that need to trigger a positive/negative shift, which need to be combined in some way to generate the overall emotional input into decision making. It may be rare for many of these factors to co-occur, so the range of emotional experience is typically well bounded and energy efficient, but in order to account for all possible factors, the capacity for feeling should they all happen to co-occur needs to be vastly higher.

Further to the counter-arguments above, we have also identified three stylized facts that suggest the HTV is likely to apply in human context: (a) empirical observations about neurological function, (b) the accounts of those who developed and apply certain pain/discomfort scales, and (c) the presence of extreme events that might prompt dramatic emotional responses.

(a) Certain empirical observations about neurological function identify patterns that are characterized by heavy tailed distributions. If these heavy-tailed neurological features extend to the neurological components of valence experience, then the HTV hypothesis is more likely. In one example, Klaus et al. (2011) found that neuronal avalanches in macaque monkeys are characterized by heavy-tailed power law distributions. It is possible that more intense experiences sometimes correspond to more intense cascades of bursts of activity in particular neuronal networks. Power laws have also been reported in spike counts (Teich et al., 1997) and ion channel fluctuations (Toib et al., 1998), potentially due to information transmission optimization features (Beggs and Plenz, 2003; albeit contested, e.g., Bedard et al., 2006; Dehghani et al., 2012). Heavy-tailed distributions of neurological activity may also translate into heavy-tailed accounts of pain experiences, such as in cluster headache frequency data (Gómez-Emilsson, 2019).

(b) Vivid accounts of the range of possible experiences can be seen in the testimony of those who created certain sensory pain scales. The KIP scale for pain intensity is recorded on a 0 to 10 scale, with the explicit instruction in the context of cluster headaches to interpret the data logarithmically: a KIP 10 is not twice as bad as a KIP 5 but 10 times as intense (Cluster Busters, 2020). Schmidt’s Sting Index reports the pain of insect skins based on personal experience on a scale that he suggests be interpreted logarithmically: “Each number is like 10 equivalent of the number before. So 10 honey bee stings are equal to 1 harvester ant sting, and 10 harvester ant stings would equal one bullet ant sting” (Peterson, 2018).

(c) Even if our own lives have been characterized by a relatively modest range of painful and pleasant events giving rise to a modest range of emotional responses, the possibility of far more dramatic events may allow us to infer proportionately more intense responses. For instance, the majority of the population who have never been physically tortured or taken heroin may nonetheless expect a truly intense experience if that were to happen.

In this paper, we suggest that the balance of stylized facts and counter-arguments point toward the HTV being true. However, there remains scope for dispute within these interpretations, meaning that empirical testing is required to establish the case either way with confidence. Given limitations in our pilot study, we would also recommend a larger and refined survey drawing on the lessons learned from this exercise.

### 5.5. Limitations and ideas for future studies

One foundational critique of our approach is whether it is reasonable to ask respondents to translate their felt experience into numerical scores. While the majority of respondents provided mathematically consistent answers, some did not, affirming the difficulty of this exercise. Self-reported measures of happiness and subjective wellbeing are widely used, but with frequent discussion of the possible limitations (see, e.g., Diener et al., 2018). Based on this experience, we suggest that useful insights can be gained from such data, even if best considered as numerical intuitions with considerable measurement error, rather than precise data. However, future surveys could take steps to help respondents engage with the method.

In this case, concerns may be exacerbated by the focus on extreme events, which may be harder to recall and analyze than evaluative wellbeing in general. We might worry that (some) respondents are hyperbolic in their responses or unable to quantify such feelings more generally, noting concerns about numeracy (e.g., Bruine de Bruin and Slovic, 2021). Acknowledging that such concerns cannot be fully alleviated, additional questions may help assess how worried we should be.

Questions could assess a propensity to hyperbole, perhaps through direct self-report, asking about how friends might describe the respondent, or asking questions that might elicit more easily tested exaggeration. Questions could similarly be designed to test someone’s ability to quantify in general and reason about ratios in other contexts. Providing examples for the ratio question may help people feel more able to give very small increases without diminishing the difference. Definitions of experience duration and emotions vs. moods may also improve consistency. Similarly, we could ask directly whether the most and second most extreme experiences are “about the same” in intensity, even if different scores are given. Alternatively, we could move away from mathematical self-report to visual reasoning, e.g., ask people to draw or select a homunculus depiction that reflects the different intensities they feel.

The comparison of most to second most intense experiences is sensitive to a number of factors, as discussed in §2.3, including respondents who may be able to recall many thousands of experiences and the possibility that they had two unusual but very similar outlier experiences. A larger survey helps increase the confidence that a small proportion of such outlier individuals would not skew the sample. Now that this pilot survey has identified initial parameters for parts of the valence distribution, future surveys could also ask respondents more directly about the likely number of equally sized steps from mildest to most intense experiences, anchored on their extrema ratios. Diagrams could be used to illustrate this abstract request, as well as interactive applets to demonstrate how draft answers play out in practice. We could also randomize whether respondents are asked to describe the ratio or the scalar scores first, in case a deliberate aim for internal consistency alters their responses, as well as randomizing question ordering more generally. A larger sample would permit investigation of potential asymmetries between pleasure and pain, the location of non-linearities in the scale, relationships with age, and types of experience.

More work could also be done to test the other three empirical predictions of HTV detailed in this paper. For instance, we can ask people about their mildest and average recalled experiences and about the most intense and most mild experiences they can imagine. Providing scenarios may help anchor the extreme events that are imaginable. We might also gain insight into the capacity question by asking respondents how they think the extreme experiences they have reported compare to how much better or worse it could get, how their experiences might compare to average human experience in their region, how their friends might have described a similar experience, and whether they feel their definition of what a “10” experience would feel like has changed over time and why.

Other foundational concerns include biases around memory validity, placing some caveats on the precision of findings while still permitting an initial directional assessment. For instance, fading affect bias suggests that negative memories tend to be forgotten more quickly than positive ones (Skowronski et al., 2014), but would still permit separate analysis of each construct. Another possibility is not remembering enough experiences to compare them, for instance being able to identify the most intense but being unsure about the second, such that it ends up being selected almost at random from the next dozen or so recalled experiences. Focusing individuals on a particular type of pleasure (such as loving relationships) or pain (such as physical damage) may make it easier to recall the salient experiences at the cost of narrower scope.

More generally, where our brains continually reconstruct past experiences to generate present-day narratives and attempt to support present-day planning, it is possible the actual valence of past experiences might either be exaggerated or downplayed over time to better suit those goals. For discussion see, e.g., Fredrickson (2000), reconstructive memory theory (Hemmer and Steyvers, 2009), and cultural influences on memory (Wang, 2020). Diary methods could be used (e.g., the ESM and DRM methods discussed earlier) to capture experiences nearer to the time, but may need to span years to have a chance of capturing peak experiences for many individuals.

Thinking more ambitiously, we would also welcome alternative methodologies for investigating this question that do not rely as strongly on self-report of recollections. For instance, correlations between neural activity and both experienced and recalled intensity, qualitative longitudinal research, self-report relative to induced peak experiences, revealed preferences, trade-off surveys, and various non-self-report measures (for discussion see Lucas et al., 2003; Mauss and Robinson, 2009; Goto and Schaefer, 2020).

## 6. Conclusion

This paper has argued, cautiously, for the Heavy-Tailed Valence (HTV) hypothesis: that the accessible human capacity for emotional experiences of pleasure and pain spans a minimum of two orders of magnitude.

Where the hypothesis applies, we have provided actionable advice to the research community. For practical measurement scale design, we suggest allowing non-integer responses or 100+ gradations and providing vivid reference points for users. For researchers, we recommend testing robustness to kinks in felt experience at the top end of the scale and conducting trade-off surveys to calibrate scale interpretation, similar to those used in QALY/DALY estimation in public health.

In quantitative support of this hypothesis, we report results from a pilot survey in which over half of respondents said that their most intense experiences were at least two times more intense than the second most intense. As such, it would only take six steps of the same magnitude between the most mild and the most extreme experiences to identify a range of capacity spanning two orders of magnitude. The evidence is only indirect, but with enough room for error that the Heavy-Tailed Valence hypothesis has some base credibility, sufficient to motivate more robust testing, especially as methodological choices were made to disfavor the HTV. Additional indirect evidence is found in simulations demonstrating that the reported data fit better to underlying heavy-tailed distributions of experience valence rather than narrow-tailed distributions. In qualitative support of this hypothesis, we have discussed three stylized facts in its favor and identified counter-arguments to six stylized facts against it.

Assessment of survey evidence and stylized facts is important for analyzing hypotheses about the capacity to experience because personal introspection may reveal orders of magnitude variation in experience for some individuals but not others. The former may believe the hypothesis, but the latter have little reason to do so and may feel doubtful about accounts from the former. For instance, a study by Holz et al. (2021) suggests that while we generally trust others’ accounts of their emotions, intense vocalizations of peak emotions are often distrusted. This paper suggests that the balance of evidence weighs in favor of the HTV, but recognizes the limited evidence so far and the importance of further research, drawing on the lessons learned from this pilot study.

In addition to measurement and analysis implications, the integer experience test demonstrates that the capacity for felt experience is not just an arbitrary or subjective choice of units. As a result, a prevailing HTV psychology also has important implications for personal and societal wellbeing ambitions. We close by briefly reflecting on these ambitions.

Recent evidence (e.g., Helliwell et al., 2023; Zimmer et al., 2023) shows most residents in high average income countries have fairly low pain prevalence (e.g., 25%) of mostly mild pain and seemingly high reported happiness (e.g., 7–8 out of 10). Organizations like the World Happiness Report and the What Works Wellbeing Centre explicitly interpret these as cardinal scales, implying there is room only for incremental improvements in the pain/pleasure components of wellbeing for the majority of residents. The numerical instinct that 8 is quite close to 10 might implicitly constrain societal ambition, focusing policy attention into different areas. However, combining our hypothesis and results with the work of Plant (2020), we identify a likely “kink” in the 0–10 measurement scale at the top end. As a result, we would make the explicit case that there is at least an additional order of magnitude of potential gains between around 8 and 10 in how the happiness scales are commonly used today. Such a “kink” leads to the opposite conclusion to mainstream think tanks; there is scope for much greater ambition than at present.

The capacity to experience likely also varies from person to person (cites in §2.1) and is likely amenable to alteration and training, as suggested by the effect of pain relief to dull emotional and even empathetic responses (Durso et al., 2015; Mischkowski et al., 2019), emotional blunting in SSRI treatments for depression (e.g., Goodwin et al., 2017), and therapeutic services both to reduce and increase the intensity of emotional experience (e.g., Engelhard et al., 2011; clinical trials on anhedonia, Phillips et al., 2022). Such context-dependency does not refute HTV. By contrast, it may make it more relevant. If our ability to train ourselves to increase the range of joy that can be experienced is much higher than we thought before, then there may be more value in investing in such effort.

A symmetrical implication applies to pain reduction. Heavy-tailed distributions of experience suggest that a large proportion of suffering might exist in the extremes. In such cases, some ethical frameworks might shift resource toward the most extreme cases of suffering. Even if it might take considerable effort to reduce someone from a suicidal 10 to a survivable 8 on a pain scale, this may outweigh improving many individuals from an annoying 6 to an ignorable 3. However, such implications are not guaranteed; they depend also on duration trade-offs, the tractability of the problem, probability of success, productivity gains for the beneficiary, and the costs of the interventions identifiable for further research.

Finally, we might briefly consider implications for individuals. As well as greater caution around avoiding extreme pains, we might consider the spectrum of pleasure. If peak events are hard to repeat more than a few times (such as parenthood), hard for individuals to influence (e.g., lottery or world cup wins), or draw on extensive build up for their emotional intensity (e.g., gaining a hard-fought promotion), there may be little we can do about it, even knowing such peak experiences exist. However, we suggest a more optimistic view, drawing inspiration from personal accounts and brain scans of activities like jhana meditation (e.g., Hagerty et al., 2013). That some experiences are hard to design for does not rule out the possibility for all experiences. We wonder what could be achieved with a more widespread attitude that peak experiences are possible, that an environment can be made more conducive to their occurrence, and that we can get better at noticing and appreciating them.

Reflecting on the link between hedonic and evaluative wellbeing, we wonder about the importance of integrating peak moments constructively into a personal life narrative. A single religious experience, with a 10/10 positive valence, may become a foundational memory for an individual, inspiring greater acts and happiness for years to come. By contrast, a single ill-timed drug experience may also have a 10/10 valence at the time, but be felt by the individual as a moment of shame and confusion in later years that they fear to experience again in case it leads to addiction; a memory to be pushed away rather than drawn upon. Could the latter be made more like the former? If life could be punctuated with many more and more incredible experiences than commonly believed, how differently would we live it for ourselves? How can we structure our collective institutions to promote, support, and leverage peak experiences?

## Data availability statement

The datasets presented in this study can be found in online repositories at: https://osf.io/udmxn/. Details: Open Science Framework Data Repository under project name Heavy-Tailed Valence Hypothesis (HTV).

## Ethics statement

The studies involving humans were approved by the Qualia Research Institute. The studies were conducted in accordance with the local legislation and institutional requirements. The participants provided their written informed consent to participate in this study.

## Author contributions

AG-E came up with the HTV hypothesis, identified intuitions for its validity, and ran the initial survey. CP developed the integer experiment test to demonstrate the hypothesis could not be collapsed into arbitrary unit mapping, identified evidence against the hypothesis, considered its validity, reviewed the literature to ascertain the originality of the argument, relationship to pre-existing research, and potential implications. AG-E and CP worked together to analyze the data and contributed to writing the manuscript and testing and refining the arguments. All authors contributed to the article and approved the submitted version.

## Footnotes

^1^Simply increasing the standard deviation of a normal distribution does not achieve this same result. As with the standardized normal distribution, the relative distributional shape is robust to linear transformations. The standard deviation can be altered arbitrarily with unit scaling without changing the underlying distribution.

^2^As an aside, we do not necessarily associate such a neutral state with having “zero” value in any ethical sense, reserving this topic for discussion elsewhere.
